# Analysis of networks in the dorsolateral prefrontal cortex in chronic schizophrenia: Relevance of altered immune response

**DOI:** 10.3389/fphar.2023.1003557

**Published:** 2023-03-23

**Authors:** América Vera-Montecinos, Ricard Rodríguez-Mias, Èlia Vila, Judit Villén, Belén Ramos

**Affiliations:** ^1^ Psiquiatria Molecular, Parc Sanitari Sant Joan de Déu, Institut de Recerca Sant Joan de Déu, Sant Boi de Llobregat, Spain; ^2^ Department of Genome Sciences, School of Medicine, University of Washington, Seattle, WA, United States; ^3^ Centro de Investigación Biomédica en Red de Salud Mental, CIBERSAM (Biomedical Network Research Center of Mental Health), Ministry of Economy, Industry and Competitiveness, Institute of Health Carlos III, Madrid, Spain; ^4^ Department de Bioquímica i Biología Molecular, Facultat de Medicina, Universitat Autònoma de Barcelona, Barcelona, Spain; ^5^ Facultat de Medicina, Universitat de Vic-Universitat Central de Catalunya, Vic, Spain

**Keywords:** DLPFC, schizophrenia, postmortem, molecular network, immune system

## Abstract

The dorsolateral prefrontal cortex (DLPFC) has a crucial role in cognitive functioning and negative symptoms in schizophrenia. However, limited information of altered protein networks is available in this region in schizophrenia. We performed a proteomic analysis using single-shot liquid chromatography-tandem mass spectrometry of grey matter of postmortem DLPFC in chronic schizophrenia subjects (n = 20) and unaffected subjects (n = 20) followed by bioinformatic analysis to identify altered protein networks in schizophrenia (PXD024939 identifier in ProteomeXchange repository). Our results displayed a proteome profile in the DLPFC of 1989 proteins. 43 proteins were found significantly altered in schizophrenia. Analysis of this panel showed an enrichment of biological processes implicated in vesicle-mediated transport, processing and antigen presentation *via* MHC class II, intracellular transport and selenium metabolism. The enriched identified pathways were MHC class II antigen presentation, vesicle-mediated transport, Golgi ER retrograde transport, Nef mediated CD8 downregulation and the immune system. All these enriched categories were found to be downregulated. Furthermore, our network analyses showed crosstalk between proteins involved in MHC class II antigen presentation, membrane trafficking, Golgi-to-ER retrograde transport, Nef-mediated CD8 downregulation and the immune system with only one module built by 13 proteins. RAB7A showed eight interactions with proteins of all these pathways. Our results provide an altered molecular network involved in immune response in the DLPFC in schizophrenia with a central role of RAB7A. These results suggest that RAB7A or other proteins of this network could be potential targets for novel pharmacological strategies in schizophrenia for improving cognitive and negative symptoms.

## 1 Introduction

The dorsolateral prefrontal cortex (DLPFC) plays a crucial role in schizophrenia (SZ) (Yoon et al., 2008). Prefrontal cortex governs executive functions (Kouneiher et al., 2009), which involve all rational thinking cognitive processes such as attention, behavior, cognitive flexibility and impulse inhibition (Logue and Gould, 2014). In the context of SZ, studies have shown cognitive, social, and emotional impairments (Russell et al., 2000; Ragland et al., 2007; Ursu et al., 2011), behaviors associated with altered functioning of the DLPFC. Evolutionarily, the prefrontal cortex is the last brain region to develop (Teffer and Semendeferi, 2012). Thus, altered cognitive functioning in SZ could be due to the dysfunction in the maturation of connectivity between the prefrontal cortex and other brain areas. Studies have demonstrated that maturation of connectivity occurs in two periods, the first during the prenatal and perinatal phases and the second during the global maturation of the circuit to establish functional networks, which lasts until early adulthood (Teffer and Semendeferi, 2012). In this regard, dysfunction in dopaminergic (Meyer-Lindenberg et al., 2002; Slifstein et al., 2015), GABAergic (Lewis et al., 1999; Tanaka, 2008) and glutamatergic (Li et al., 2019) neurotransmission systems has been reported in the prefrontal cortex in SZ. Thus, altered neuronal networks underlie SZ and several hypotheses could be unified to understand this dysfunction.

Altered expression of genes associated to immune system (Arion et al., 2007) and inflammatory response (Fillman et al., 2013) has been described in DLPFC in SZ. Genetic predisposition together with insults during the perinatal such as maternal infection could lead to the accumulation of errors during neurodevelopment. Dysfunction of the immune system is one of the hypotheses that underlie SZ, which proposes that maternal infection during the gestational period could increase the risk for SZ in offspring exposed to viral or bacterial infections in intrauterine life (Brown et al., 2004). In this regard, high levels of IL-8 during pregnancy have been implicated in a decrease in brain volume and an increase volume of ventricles in schizophrenia subjects (Ellman et al., 2010). Cytokines such as Interferon-Ƴ can modulate and inhibit the differentiation of oligodendrocytes and alter myelinization processes (Chew et al., 2005). Thus, inflammatory processes could alter myelinization and lead to neuronal connectivity dysfunction. The involvement of immune dysfunction and inflammation in SZ is supported by emerging evidence suggesting aberrant immune mechanisms (O’Donnell et al., 1995; Kroken et al., 2019; Comer et al., 2020; Afia et al., 2021). A genome-wide association study reported a major histocompatibility complex (MHC) locus and Toll-like receptors (TLRs), both involved in innate immunity, as genetic risks for SZ (Bramon et al., 1038). Indeed, postmortem studies of SZ subjects showed altered expression of elements of TLR4 signaling (García-Bueno et al., 2016; MacDowell et al., 2017). Moreover, MHC has several functions in the central nervous system during development including neurite outgrowth and neuronal plasticity (Shatz, 2009; Elmer and McAllister, 2012). The MHC II has a role in antigen presentation during adaptative response (Shatz, 2009). In this context, studies have demonstrated altered immune components in SZ subjects such as a decrease of dendritic cells, involved in the antigen presentation (Fernandez-Egea et al., 1556), an increase in the ratio of lymphocytes/neutrophils, an important marker for inflammatory damage (Karageorgiou et al., 2019; Sandberg et al., 2021) and increase in monocytes (Jackson and Miller, 2020) which is associated with an increase of proinflammatory cytokines in blood (Kowalski et al., 2001). Altered signaling during the immune response could lead to the release of proinflammatory molecules. Studies in postmortem DLPFC have found high mRNA levels of proinflammatory cytokines in this area, including IL1β, IL6, IL8, and IL 1 in SZ subjects (Fillman et al., 2013; Cai et al., 2020)]. Recently, studies have suggested the existence of two sub-biotypes for SZ, based on the levels of proinflammatory molecules in the peripheral blood sample which could reflect the state of the central nervous system. The SZ subjects with high levels of proinflammatory cytokines have difficulty with language and decreased cortical volume (Fillman et al., 2016; Bowen et al., 2019; Childers et al., 2022). These molecules would alter cerebral barriers and lead to molecules entering the cerebral parenchyma, thus altering the homeostatic balance in the brain and contributing to the global dysregulation of neuronal networks in SZ. All this evidence supports that the activation of the immune response is performed by a complex network of molecular interactions and the dysfunction of this complex network could be responsible for the inflammatory processes in DLPFC in SZ.

The proteomic approaches allow to investigate protein signatures and molecular mechanisms involved in specific brain areas in SZ. The aim of our study was to identify altered protein networks in the DLPFC of SZ patients using single-shot liquid chromatography-tandem mass spectrometry analysis. In this study, we compared the proteomic profile in postmortem DLPFC (Brodmann area 9) of individuals with chronic SZ (n = 20) and unaffected subjects (n = 20). Our results showed a downregulated network involved in the immune response in DLPFC in which RAB7A protein has multiple interactions in this network.

## 2 Materials and methods

### 2.1 Postmortem prefrontal cortex brain tissue

Samples from dorsolateral prefrontal cortex of subjects with chronic SZ (n = 20, left hemisphere), and unaffected subjects (n = 20, 10 left hemispheres and 10 right hemispheres). The samples of chronic SZ were obtained from the collection of neurologic tissue of *Parc Sanitari Sant Joan de Déu* Brain Bank (Roca et al., 2008). For unaffected subjects the samples of prefrontal cortex were obtained from the Institute of Neuropathology of Hospital *Universitari de Bellvitge*, Navarra Biomed Biobank and *Hospital Clinic* IDIBAPS Biobank. The brain banks of Parc Sanitari Sant Joan de Déu, Institute of Neuropathology of Hospital *Universitari de Bellvitge, Hospital Clinic* IDIBAPS Biobank follow the same protocol based on the guidelines from the Network of European Brain and Tissue Banks for Clinical and Basic Neuroscience (BrainNET Europe). According to their standardized protocols, fresh brain hemisphere was cut in coronal slabs of 1 cm of thickness, frozen in dry ice (−20°C) and stored at −80°C. In Navarra Biomed Biobank, fresh brain tissue was immersed into isopentane over nitrogen liquid at −180°C for rapidly freeze and were stored at −80°C. Human Brodmann area nine from DLPFC was dissected from coronal slabs stored at −80°C extending from the pial surface to white matter only including grey matter. pH was measured using pH indicator strips (Merk, range pH 5–10) in all the brain samples immediately before performing the dissection of brain tissue for protein extraction. All SZ subjects were institutionalized donors with a long duration of the illness who had no history of neurological episodes. We matched SZ and control groups by gender (only male patients were included), age, postmortem delay (PMD) and pH (Table 1). Experienced clinical examiners interviewed each donor *antemortem* to confirm SZ diagnosis according to the Diagnostic and Statistical Manual of Mental Disorders (DSM-IV) and International Classification of Diseases 10 (ICD-10). Besides, donor subjects were evaluated *antemortem* with Positive and Negative Syndrome Scale (PANSS) and for neurocognitive measures was used the Frontal Assessment battery (FAB) with a death to clinical assessment interval of 61 months. All deaths were due to natural causes. The study was approved by the Institutional Ethics Committee of Parc Sanitari Sant Joan de Déu. A written informed consent was obtained from each subject. The last daily chlorpromazine equivalent dose for the antipsychotic treatment of patients was calculated based on the electronic records of last drug prescriptions administered up to death as described previously (Gardner et al., 2010).

**TABLE 1:** Demographic, clinical and tissue-related features of cases.

	Schizophrenia (n = 20)	Control (n = 20)	Statistic	*p*-value
Gender (Male)	100% (n = 20)	100% (n = 20)	N/A	N/A
Age (years)	74 ± 10	74 ± 9	0.06; 38^a^	0.94
PMD (hours)	5.15 ± 2.59	6.07 ± 2.81	1.07; 38^a^	0.29
pH PFC	6.76 ± 0.75	6.67 ± 0.48	0.42; 38^a^	0.67
SZ diagnosis			N/A	
Chronic residual	70% (n = 14)			
Chronic paranoid	15% (n = 3)			
Chronic disorganized	5% (n = 1)			
Chronic catatonic	5% (n = 1)			
Simple-type	5% (n = 1)			
Age of onset of SZ (years)	23.9 ± 10	N/A		
Duration of illeness	50 ± 11	N/A		
Toxicology		N/A		
Daily AP dose (mg/day)^b^	683.9 ± 925.9			
First generation AP	20% (n = 4)			
Second generation AP	40% (n = 8)			
First and Second generation AP	25% (n = 5)			
AP free	15% (n = 3)			

Mean ± standard deviation; PMD, postmortem delay; SZ, schizophrenia; AP, antipsychotics; N/A, not applicable.

^a^ Mann-Whitney U for non-parametric variables.

^b^ Last daily chlorpromazine equivalent dose was calculated based on the electronic records of drugs prescriptions of the patients as described (Gardner et al., 2010).

### 2.2 Label free quantification proteomic analysis mass spectrometry

#### 2.2.1 Protein reduction, alkylation, LysC digestion, and desalting

Protein extracts were prepared from tissue samples using buffer lysis containing ammonium bicarbonate 50 mM, B-glycerophosphate 1M, sodium orthovanadate 100 mM, sodium pyrophosphate 200 mM, sodium fluoride 200 mM, tris 100 mM and Rapi Gest surfactant (Waters). Protein concentration was determined by Bradford assay (Biorad, Hercules, CA, United States). A total amount of 200 µg of protein per each brain protein extract from unaffected subjects and SZ subjects was used for single-shot liquid chromatography-tandem mass spectrometry (LC-MS/MS) analysis. The lysate was reduced with 3 mM dithiothreitol at 55°C for 30 min and alkylated with 20 mM iodoacetamide at room temperature for 30 min and quenched with additional 10 mM dithiothreitol for 30 min at room temperature. Peptide samples for mass spectrometer analysis were prepared from reduced and alkylated lysate by adapting a single‐pot solid‐phase enhanced sample preparation (SP3) similar to Hughes et al. (2014) and using a KingFisher™ Flex (Thermo Fisher) automated robotic magnetic bead handler (Leutert et al., 2019).

Specifically, 50 µg of lysate protein was diluted to 0.5 mg/mL concentration in 80% EtOH and loaded onto 500 µg of conditioned magnetic carboxylated sp3 beads. Beads were subsequently washed 3 times with 100 µL of 80% EtOH prior to on bead digestion/elution using 75 µL of 50 mM AMBIC buffer and LysC at 10 ng/µL. Digestion was carried out for 4 h at 37°C, beads were further washed with 75 µl of water and wash was combined with eluate. Eluate was acidified with formic acid and freeze dried prior to resuspending in MS sample buffer. Peptides were resuspended in 3% formic acid and 4% acetonitrile in water at a 1 µg/µL concentration.

#### 2.2.2 Liquid chromatography coupled to tandem mass spectrometry

The LC/MS-MS analysis was performed in Q-Exactive instrument (Thermofisher Scientific, CA, United States) using data-dependent Top 20 acquisition method. Peptides were loaded onto a 100 μm ID × 3 cm precolumn packed with Reprosil C18 3 μm beads (Dr. Maisch GmbH), and separated by reverse‐phase chromatography on a 100 μm ID × 30 cm analytical column packed with Reprosil C18 1.9 μm beads (Dr. Maisch GmbH). A gradient of 3%–30% acetonitrile in 0.125% formic acid was used delivered at 225 nL/min over 130 min, with a total 180-min acquisition time. Peptides were analyzed online on the orbitrap mass analyser using a top 20 data-dependent acquisition with all MS spectra being acquired and stored in centroid mode. Full MS scans were acquired from 300 to 1,500 m/z at 70,000 FWHM resolution with a fill target of 3E6 ions and maximum injection time of 100 ms. The 20 most abundant ions on the full MS scan were selected for fragmentation using 2 m/z precursor isolation window and beam-type collisional-activation dissociation (HCD) with 26% normalized collision energy. MS/MS spectra were collected at 17,500 FWHM resolution with a fill target of 5E4 ions and maximum injection time of 50 ms. Fragmented precursors were dynamically excluded from selection for 35 s.

**FIGURE 1:**
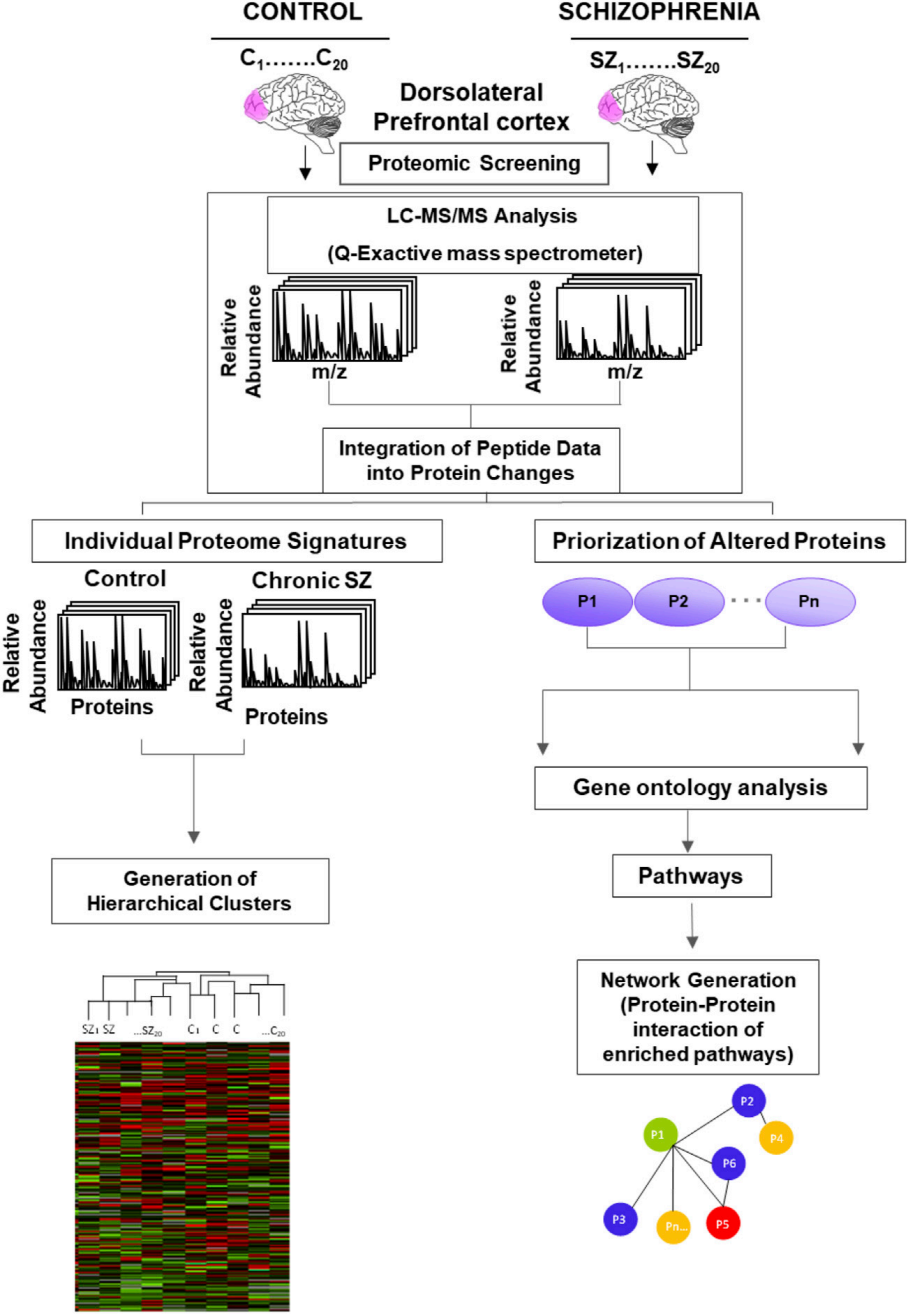
Experimental design of the proteomic analysis to identify altered pathways in schizophrenia. Protein lysates from the postmortem dorsolateral prefrontal cortex of control subjects (C) (n = 20) and chronic schizophrenia (SZ) patients (n = 20) were processed as depicted. The peptides were separated and analyzed by Liquid Chromatography (LC) coupled to a Q-Exactive Orbitrap tandem mass spectrometer. The relative fold change of peptides was integrated into protein changes. The individual protein signatures for each case and control were used to generate hierarchical clusters. The prioritization of altered proteins (P1-Pn represents generic proteins) in SZ was obtained by comparing protein fold changes between C and SZ groups (significant proteins adjusted to an FDR<0.1).

Raw files were processed and analyzed by MaxQuant (version 1.6.2.10). MS/MS spectra were searched with Andromeda against the uniprot_UP000005640_20180526. fasta with common contaminants added. The precursor mass tolerance was set to 7 ppm, and the fragment ion tolerance was set to 20 ppm. Search parameters included fully LysC enzyme specificity with up to three missed cleavages permitted. The minimum required peptide length was seven residues. The target-decoy database search strategy was used to guide filtering and estimate False Discovery Rate (FDR). Peptides matches were filtered to ≤0.01 FDR. Proteins with at least one peptide were considered identified. Label free quantification (LFQ) was selected for individual protein comparisons between control and SZ groups. A quality cut off for protein determination was the presence of the protein in the 20 samples per group, thus, proteins no quantified in all the 20 samples per group were excluded from the analysis. The normalized LFQ intensity was referred to media of the controls. A significance value for each quantified protein was calculated from Student’s t-test and correction of significance values of the quantified protein data set was performed following the Benjamini and Hochberg methods (Benjamini and Hochberg, 1995). A False Discovery Rate (FDR) was computed for all significant values and FDR threshold was set to 0.1. The experimental strategy used in this proteomic study is shown in Figure 1. The quantified proteins were imported into Perseus software platform (version 1.6.1.3) to check data quality obtained by LC/MS/MS and to visualize the data distribution (Tyanova et al., 2016). We performed four analyses for these altered proteins in SZ: i) Correlation matrix: the normalized LFQ intensity data were used to estimate a correlation coefficient matrix between controls and SZ patients. The Pearson correlation coefficients were used to defined unsupervised hierarchical cluster analysis and were plotted as a heat map; ii) Unsupervised hierarchical clustering analysis generated from quantified proteins in 20 SZ and 20 healthy control samples of postmortem DLPFC. This was carried out on Z-score transformed normalized LFQ intensity data for each protein using Euclidean distance; iii) Enrichment analysis of biological processes and pathways; and iv) Generation of networks from significantly enriched pathways by protein-protein interaction.

### 2.3 Bioinformatic analysis

To analyze the proteome profile of DLPFC we performed Disease term, Gene Ontology (GO), and Pathways analysis using Webgestalt (WEB-based GEne SeT AnaLysis Toolkit). The enrichment analysis, we used the method of Over-Representation Analysis (ORA) supported by Fisher’s exact test (Wang et al., 2013). To perform the enriched analysis, we used the whole genome as a reference set. Diseases terms were obtained from PharmacoGenetics Knowledge Base (PharmGKB) and GLAD4U Gene List Automatically Derived For You (GLAD4U) databases (Hewett et al., 2002; Jourquin et al., 2012). For GO analysis, we performed non-redundant enriched categories analysis, for pathways analysis we used the Reactome database. Multi-testing correction was performed using Benjamini–Hochberg test (FDR = 0.1). For network generation, we used String version 11.0. For screening protein localization in different cell types in cerebral cortex tissue (Endothelial cells, glia Cells and Neuronal Cells) we used the Human Protein Atlas database (Uhlén et al., 1979). The enrichment analyses were set to FDR = 0.1. For the representation of biological processes and pathways enriched we used the Log_2_ of the fold change of normalized LFQ Intensity between SZ and controls samples and FDR adjusted -Log_10_ (*q*-value).

## 3 Results

### 3.1 Quantitative proteomic analyses in the dorsolateral prefrontal cortex in chronic schizophrenia

**FIGURE 2:**
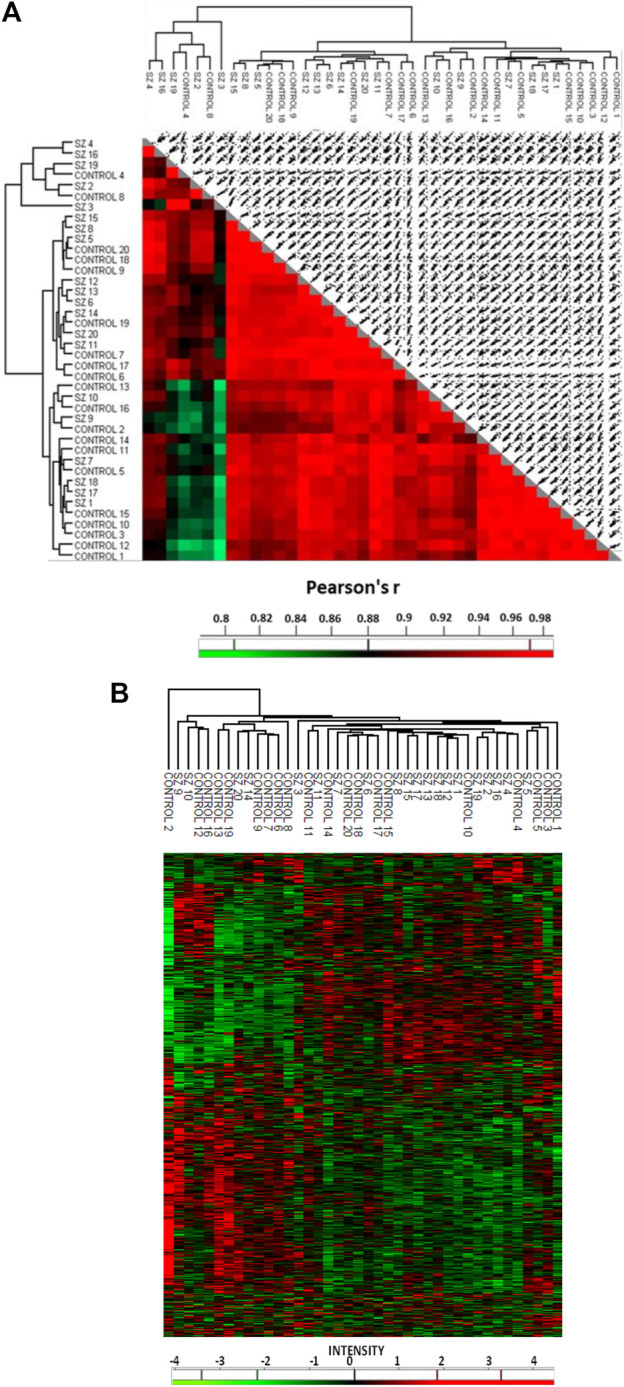
Quantitative proteomic analysis in the prefrontal cortex in schizophrenia. **(A)** The correlation matrix shows the correlation coefficiency between the 1989 quantified proteins in each compared pair of samples. **(B)** Unsupervised hierarchical clustering analysis from prefrontal cortex proteins. This analysis was performed with matrix processing according to the Euclidean distance and z-score aggregation method. We used 1989 quantified proteins. Green color clusters represent downregulated proteins. Red color clusters represent upregulated proteins. The intensity scale represents the z-score, positive scores show significantly upregulated proteins labeled in red, while negative scores significantly downregulated proteins are labeled in green. **SZ**, schizophrenia; **C**, control.

To identify altered proteins related to SZ in the DLPFC, we used individual protein extracts from a cohort of SZ subjects (n = 20) and unaffected subjects (n = 20). No differences were observed between the SZ and control groups for any demographic or tissue-related variables (Table 1). Our proteomic analysis in the DLPFC revealed a total of 4,407 quantified proteins. We excluded from the analysis 2,418 proteins that were not quantized in all the 20 samples per group. 1989 common proteins were quantified (45%) in all the 20 samples per group and used for subsequent bioinformatics analyses (Supplementary Data S1). The proteomic analysis revealed that 99.9% of the proteins were identified with two or more peptides and 91.3% with five or more peptides. We found only 12 proteins significantly altered using FDR <0.05 (Data not shown). In addition, our analysis using FDR<0.1, showed 43 significantly altered proteins in the DLPFC (Supplementary Data S2) of which 9 were upregulated and 34 were downregulated. Altered proteins were classified according to their cell type protein expression in human cerebral cortex. 28 proteins have been previously reported to have medium-high level expression in cortical neuronal cells, while only 10 proteins in glia cells and 11 proteins in endothelial cells (Supplementary Data S2). We further classified the altered proteins according to their abundance in excitatory and inhibitory neurons. Only SYNJ1, TXNRD1 and NEFM were found to have different expression levels between both neuronal types. To analyze the possible influence of tissue-related features on the altered proteins, we performed correlation analysis in all samples. None of the regulated proteins showed significant correlation with PMD or pH (FDR<0.1) (Supplementary Datas S3, S4). Further, no significant correlation was observed between altered proteins and antipsychotic dose (Data not shown). 42 of the 43 altered proteins had been previously reported in gene expression analyses in SZ from the Schizophrenia Database and 12 of the 43 altered proteins had been previously reported in proteomic studies (Supplementary Data S2). For the individual proteome signature analysis, we first examined the similarity of the individual proteome in the DLPFC through a correlation matrix (Figure 2A). The results showed correlations above a Pearson correlation coefficient of 0.7 with an average coefficient of 0.994 between the SZ and control samples indicating a high quality of the proteomic data. Although two main hierarchical clusters were identified in this analysis, we did not find any clinical or tissue-related feature that explain the segregation of these two clusters. We then analyzed any possible laterality effect on these results. However, we did not find any significant differences between the right (n = 10) and left (n = 10) hemispheres in the control group in our study and no segregation of clustering analysis by hemisphere was observed (Supplementary Figure S1 and Supplementary Data S5). In addition, the brain biobank source of each sample in the unaffected group of subjects was analyzed in this clustering analysis. No segregation by center was observed (Supplementary Figure S1). The unsupervised hierarchical clustering analysis revealed that the proteomic profile detected for each subject did not allow segregation between controls and SZ group (Figure 2B).

### 3.2 Gene ontology enrichment analysis for biological processes and pathways

**FIGURE 3:**
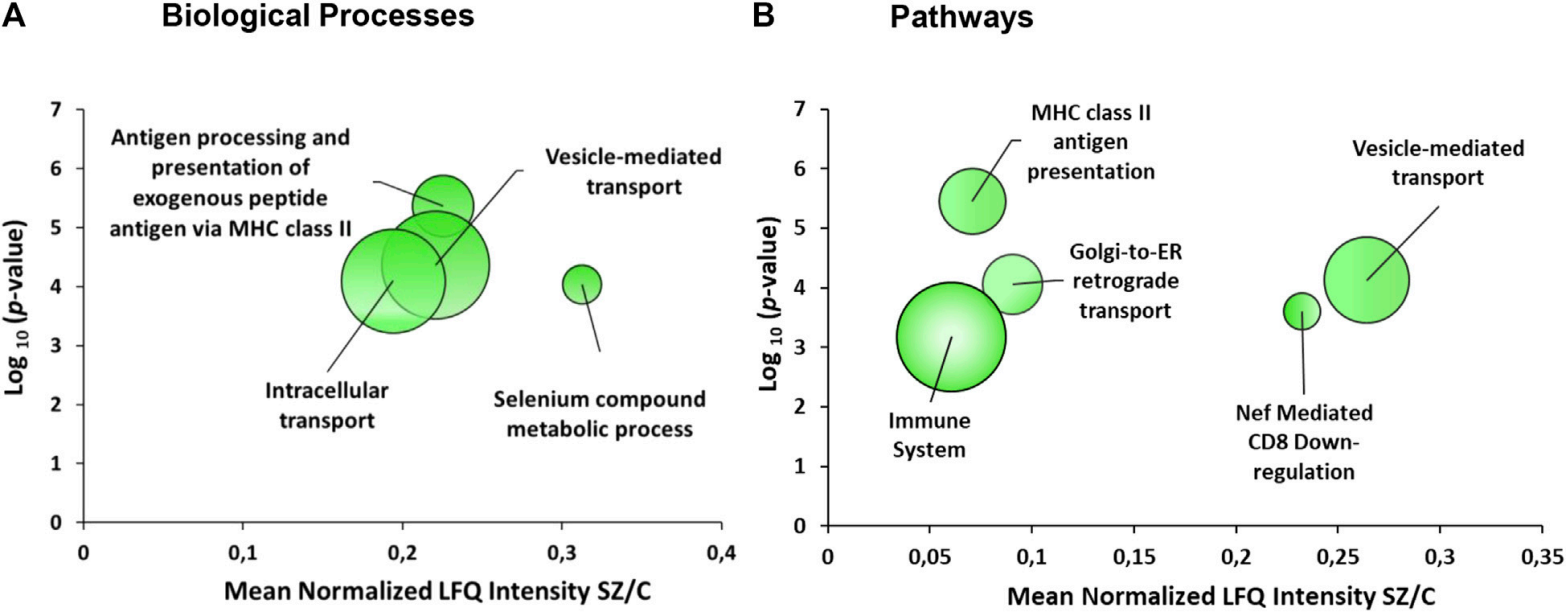
Enrichment analyses from the dorsolateral prefrontal cortex proteome in chronic schizophrenia. The bubble chart shows enriched categories from 43 altered proteins in schizophrenia. **(A)** Non-redundant enriched biological processes categories were: antigen processing and presentation of exogenous peptide antigen *via* MHC class II (GO:0019886), vesicle-mediated transport (GO:0016192), intracellular transport (GO:0046907) and selenium compound metabolic process (GO:0001887). **(B)** The enriched pathway categories in schizophrenia were: MHC class II antigen presentation (R-HSA-2132295), vesicle-mediated transport (R-HSA-5653656), Golgi-to-ER retrograde transport (R-HSA-8856688), Nef-mediated CD8 downregulation (R-HSA-182218) and immune system (R-HSA-168256). The *X*-axes show the mean of normalized LFQ intensity in schizophrenia relative to the control group for all the proteins that belonged to each category. The *Y*-axes show the -log_10_ enrichment *p*-value. The bubble size is directly proportional to the number of proteins represented in each enriched category, biological process or pathway. The green color represents downregulated proteins. **SZ**, schizophrenia; **C**, control; **LFQ**, label free quantification.

The gene ontology analysis with the 43 proteins that were altered in the DLPFC showed an enrichment of several biological processes (FDR = 0.1) (Figure 3A; Table 2) involved in vesicle-mediated transport, processing and antigen presentation *via* MHC class II, intracellular transport and selenium metabolism. The enriched pathways (Figure 3B) were directly related to the enriched biological processes observed. These pathways were related to MHC class II antigen presentation, vesicle-mediated transport, Golgi transport and immune system. All these enriched pathways were found for the downregulated proteins.

**TABLE 2:** Non-redundant gene ontology analysis of the 43 proteins altered in the dorsolateral prefrontal cortex.

Classification	Category	Proteins overlap in category	Total number	Observed number	E	*p*-value	FDR
**BIOLOGICAL PROCESSES**	Antigen processing and presentation of exogenous peptide antigen *via* MHC class II	ACTR1A; AP2M1; AP2S1; DCTN5; RAB7A	97	5	0.24	4.32 × 10^−6^	0.02
	Vesicle-mediated transport	RAB6B; SYNJ1; ACTR1A; PLCG1; AP2M1; ACLY; AP2S1; ATG7; ARF3; DCTN5; GPI; RAB5C; CYFIP2; LIN7C; RAB7A	1942	15	4.89	4.28 × 10^−5^	0.06
	Intracellular transport	RAB6B; RAN; SYNJ1; ACTR1A; AP2M1; AP2S1; ARF3; PPP1CC; NEFL; DCTN5; RAB5C; TIMM10; SRSF9; RAB7A	1803	14	4.54	8.06 × 10^−5^	0.08
	Selenium compound metabolic process	SCLY; TXNRD1	6	2	0.02	9.24 × 10^−5^	0.08
**PATHWAYS**	MHC class II antigen presentation	ACTR1A; AP2M1; AP2S1; TUBB3; DCTN5; RAB7A	121	6	0.42	3.55 × 10^−6^	0.01
	Vesicle-mediated transport	ACTR1A; SYNJ1; RAB6B; AP2M1; AP2S1; TUBB3; ARF3; DCTN5; RAB5C; RAB7A	667	10	2.33	7,39 × 10^−5^	0.03
	Golgi-to-ER retrograde transport	ACTR1A; RAB6B; TUBB3; ARF3; DCTN5	131	5	0.46	8.72 × 10^−5^	0.04
	Nef Mediated CD8 Downregulation	AP2M1; AP2S1	7	2	0.02	2.47 × 10^−4^	0.08
	Immune System	ACTR1A; PLCG1; PRKACA; AP2M1; ACLY; ATP6V1E1; AP2S1; TUBB3; ATG7; TRIM2; GPI; CYFIP2; LMNB1; DCTN5; RAB5C; RAB7A	1997	16	6.98	6.63 × 10^−4^	0.09

For the gene ontology analysis, we used the Gene Ontology Consorcium database: pathways were identified according to the Reactome database and proteins assigned to each pathway are listed. Total number: number of reference proteins in the category/pathway. Observed number: proteins in the data set and also in the category/pathway. E: expected in the category. Adjusted *p*-value is corrected for multiple test, FDR<0.1. These analyses were carried out using Webgestalt. the reference set was the whole genome.

### 3.3 Network generation from enriched pathways in the dorsolateral prefrontal cortex

**FIGURE 4:**
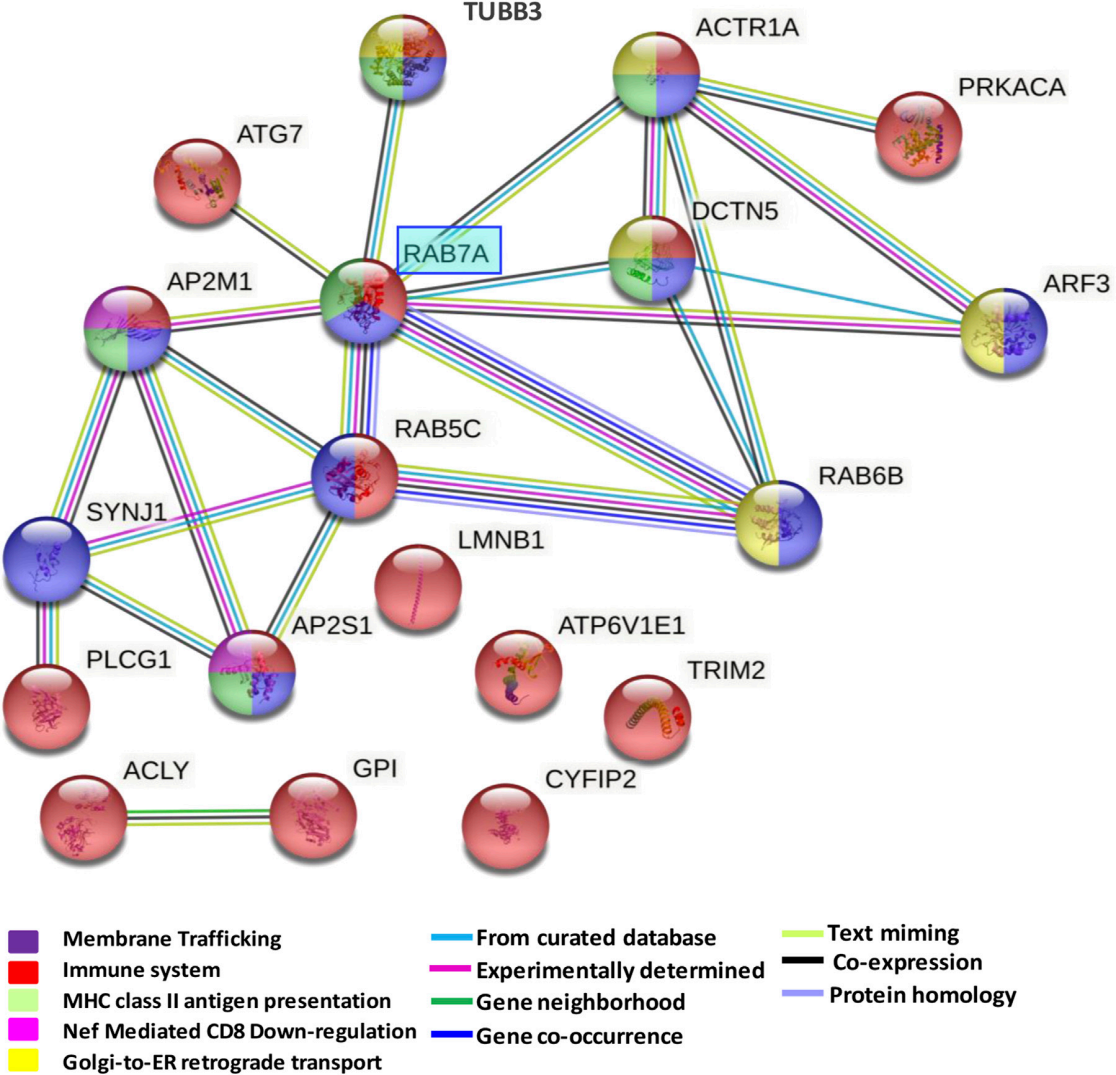
Network formed by altered pathways in the dorsolateral prefrontal cortex in schizophrenia. The protein-protein interaction network illustrates a mixed module with interactions and overlaps among proteins formed by downregulated pathways. The interaction overview shows how proteins overlap in the different pathways. Each node represents a protein. The color denotes membership of a module. The colored edge (connections between nodes) represents the type of interaction between nodes. The network was generated using String V.11.

Network analysis of significantly enriched pathways from the 43 proteins altered in the dorsolateral prefrontal cortex showed an overlap among the several altered pathways (Figure 4). A mixed module was observed in which the main overlap was generated by the immune system pathway. This pathway showed about 43% overlap with membrane trafficking proteins, 37% with MHC class II antigen presentation, 12% with Nef-mediated CD8 downregulation and 31% with Golgi ER retrograde transport. All 13 proteins of the network were proteins with known expression in cortical neurons being eight of them with medium to high expression levels (Supplementary Data S2). Furthermore, RAB7A showed eight interactions with proteins involved in all enriched pathways.

### 3.4 Correlation analysis between proteins altered in the dorsolateral prefrontal cortex and executive function and negative symptoms

We performed a correlation analysis between the protein levels and the Frontal Assessment battery (FAB) and negative symptom scores. We did not find any significant correlation between proteins altered in the DLPFC and FAB scale, which measures only executive function. However, we found a significant correlation between negative symptom scale and Cytoplasmic FMR1-interacting protein 2 (CYFIP2) protein (r = −0.738, FDR< 0.1). CYFIP2 showed a significant association with some specific domains of negative symptom scale: blunted affect (r = −0.625, FDR< 0.1), passive/apathetic social withdrawal (r = −0.666, FDR< 0.1), and lack of spontaneity and flow of conversation (r = −0.781, FDR< 0.1).

## 4 Discussion

Our study has allowed us to identify and characterize the altered proteome profile in the DLPFC in chronic SZ. We identified a panel of 43 altered proteins in this region. 65% of the proteins belong to proteins with medium-high expression levels in neurons indicating a higher impact on neuronal functioning. The enriched altered pathways were mainly related to the immune system. The network generated from the enriched pathway showed a mixed module with interactions between MHC class II antigen presentation, membrane trafficking, Golgi-to-ER retrograde transport, Nef-mediated CD8 downregulation and the immune system. All altered proteins of the network were proteins with known expression in cortical neurons being more than half of them with medium to high levels in this cell type. RAB7A showed eight interactions with proteins of all these pathways. Thus, our results provide an altered molecular networks involved in immune response in the DLPFC in schizophrenia with a central role of RAB7A.

### 4.1 Network generation from pathways enriched in the dorsolateral prefrontal cortex

The immune system is essential for the correct maintenance of brain homeostasis. Our results from the proteome of the DLPFC showed enrichment for biological processes and pathways related mainly to the immune response. It is well known that intrauterine or perinatal infections can increase vulnerability to SZ (Brown and Derkits, 2010). An imbalance in the immune system has been proposed as one possible hypothesis that underlies this disorder (Kinney et al., 2010). Thus, our findings support an imbalance in the immune system in the prefrontal cortex in schizophrenia.

#### 4.1.1 Major histocompatibility complex class II in antigen presentation

The immune response involves major histocompatibility complex class II antigen presentation (MHC-complex class II). This process is essential in the adaptative immune response (Bondy, 2020). The MHC class II molecule is assembled in the endoplasmic reticulum, transported to the Golgi apparatus and subsequently carried by the endocytic pathway to late endosomes and lysosomes where the MHC class II complexes are loaded with peptides from antigens and sent to the cell surface (Hiltbold and Roche, 2002). MHC II has been proposed as a risk gene for SZ (Consortium, 2009). In this context, our study identified an altered MHC-complex class II antigen presentation pathway in the prefrontal cortex in SZ. Our analysis identified proteins that were altered in this pathway such as AP2M, which is involved in MHC II molecule trafficking (McCormick et al., 2005), a process is crucial in antigen presentation. In line with our study, a previous proteomic study has also reported altered levels of AP2M in the anterior cingulate cortex in SZ (Föcking et al., 2015). Thus, our study provides additional evidence of a disruption in antigen presentation mediated by MHCII in SZ.

#### 4.1.2 Golgi membrane trafficking in immune response: A central role of RAB7A

Our study identified an alteration of RAB7A in the prefrontal cortex with multiple interactions with other altered proteins from the network with a role on dynactin complex formation for motility of Golgi-associated vesicles. Thus, altered RAB7A could be impacting on different processes of immune response related to Golgi-mediated trafficking in this brain area in SZ.

RAB7A is a Rab GTPase that is involved in the regulation of degradative signaling of autophagosomes (Gutierrez et al., 2004). During infection, the microorganism is internalized into the autophagosome, which fuses with lysosomes for the degradation of its contents (Harrison et al., 2003). Altered expression of RAB7A in prefrontal cortex could lead to the dysfunction of the degradation process, thereby contributing to dysregulation in the innate and adaptive response in SZ.

Our analysis also found altered ACTR1A protein in the prefrontal cortex in schizophrenia. This protein is an actin-related protein that participates in the dynactin complex (Eckley et al., 1999). A study associated ACTR1A with the regulation of Toll-like receptor 2 (TLR2) signaling (Re and Strominger, 2001). This signaling has an active function in the innate immune response and the recognition of microbial products in dendritic cells in the brain. However, there is no evidence of the role of ACTR1A in the adaptative immune response. Moreover, ACTR1A could interact with DCTN5, another altered protein in our study. Both proteins are involved in the dynactin complex (Urnavicius et al., 1979; Eckley et al., 1999), which is involved in the activity of dynein during several intracellular motility processes (Jaarsma and Hoogenraad, 2015). Dynactin is recruited to the Golgi membrane by RAB6B and RAB7A GTPases, which contribute to the motility of Golgi-associated vesicles (Short et al., 2002). Here these two proteins, RAB6B and RAB7A, were found altered in the prefrontal cortex in schizophrenia with an interaction with both ACTR1A and DCTN5. In this context, we found altered pathways related to vesicle-mediate transport and Golgi-to-ER retrograde transport. Altered vesicle-mediated transport could be a reflection of the disruption in RAB6B and RAB7A, proteins that are altered in this pathway that affect the recruitment of dynactin to the Golgi membrane. These pathways could be directly related to the immune system, which could generate the dysfunction in the several steps involved during the immune response in SZ subjects. The Golgi apparatus has recently been proposed as a signaling station that facilitates processes related to the innate immune response (Chen and Chen, 2018) and the interface between the Golgi, endoplasmic reticulum and mitochondria has been proposed to form an activity core in inflammasome activation (Chen and Chen, 2018). Furthermore, a study has shown that palmitoylation in the Golgi apparatus is an essential step for activation of interferon genes in response to genetic pathogen material (Mukai et al., 2016). In this regard, studies in the postmortem prefrontal cortex in SZ subjects also identified an altered glycosylation process in the Golgi apparatus (Bauer et al., 2010; Mueller et al., 2014). Thus, our study showed altered Golgi apparatus functioning related to the dysfunction in the immune response in SZ.

#### 4.1.3 The dysregulation of Golgi-to-ER retrograde transport in synaptic function

The synapse function depends on correct intracellular membrane trafficking (Liu and Storrie, 2012). Our study shows the dysregulation of Golgi-to-ER retrograde transport in DLPFC. Thus, the dysfunction of Golgi-to-ER retrograde transport could contribute to altered membrane trafficking. For normal membrane trafficking, several proteins are finely regulated. In this context, the Rab proteins, SNAREs, and COP proteins are essential to regulate the trafficking of proteins and lipids involved in the neuronal structure and synaptic function (Liu and Storrie, 2012; Ridvan Kiral et al., 2018). In this line, our study found downregulation of RAB6B. This protein has a key role in the Golgi-to-ER retrograde transport (Zhang et al., 1493; Lu et al., 2021). Furthermore, the altered Golgi-to-ER retrograde transport could be due to Golgi fragmentation leading to altered membrane trafficking, sphingomyelin biosynthesis (Rasika et al., 2019), transport to axons and dendrites (Pottorf et al., 2018), and dendrite growth and branching (Wang et al., 2020). The Golgi complex is also essential for glycosylation. In this context, a recent study in SZ has shown abnormal glycosylation of AMPA receptors which are involved in the postsynaptic response in excitatory signaling (Mueller and Meador-Woodruff, 2020). The abnormal glycosylation could be due to the dysfunction of the Golgi complex. Altered membrane trafficking could lead to the existence of synaptic abnormalities which have been previously documented in SZ (Kao and Porton, 2009; Hall et al., 2020). Several studies including genetic and proteomic studies have reported a disruption in the expression of synaptic vesicle proteins in this disorder (Pennington et al., 2008; Fromer et al., 2014; Egbujo et al., 2016; Onwordi et al., 2020). These deficits have been also observed in the prefrontal cortex (Yan and Rein, 2021). This region is very sensitive to neurochemical changes (Arnsten, 2009), and dysfunction in synaptic transmission in this area could impact prefrontal cortex function. Thus, altered Golgi-to-ER retrograde transport in SZ could alter the synaptic function through the disruption of vesicle-mediated neurotransmitter release in the presynaptic terminal and the post-translational modification of AMPA receptors in the postsynaptic membrane, which could lead to altered of neuronal connectivity in this disorder.

#### 4.1.4 Nef mediated CD8 downregulation

Here we also found enriched Nef-mediated CD8 downregulation pathway in prefrontal cortex in schizophrenia. Nef (Negative regulatory factor) is a protein that enhances viral particle infectivity (Sacha et al., 2007) CD8^+^ is a molecule present in T cells that is related to the immune response against infected cells mediated by MHC I (Smolders et al., 2018). Thus, the Nef-mediated CD8 downregulation mechanism is interfering with the ability of CD8^+^ T cells to inhibit viral replication causing a depressed immune response to the viral infections (Palesch et al., 2020). Aberrant immune response during viral infection could induce the infection of glial cells and the activation of astrocytes with subsequent release of proinflammatory molecules and reactive oxygen species (Borrajo et al., 2021) and contributing to neuronal damage in the central nervous system. Furthermore, Nef is transported in extracellular vesicles (EVs) during viral infection. These EVs could be transported from astrocytes into neurons which could become more vulnerable to viral infection (Sami Saribas et al., 2017). Nef could also inhibit the EVs formation from CD4^+^ T cells which could alter the recognition of circulating microbes (Borrajo et al., 2021). Thus, dysfunction of the immune response to viral and bacterial infections mediated by Nef could be responsible for the ability of microbes to induce injuries in the DLPFC contributing to an altered functioning of this region in SZ.

### 4.2 Altered expression of CYFIP2 in SZ

Here we found that CYFIP2 is downregulated in prefrontal cortex in SZ. We found a significant correlation between CYFIP2 and negative symptoms in our SZ cohort. Negative symptoms are the most resistant to currently available pharmacological interventions in SZ (Correll and Schooler, 2020; Malashenkova et al., 2021). Negative symptoms in PANSS include seven specific domains, including blunted affect, emotional withdrawal, poor rapport, passive/apathetic social withdrawal, difficulty in abstract thinking, lack of spontaneity and flow of conversation, and stereotyped thinking (Shankar and Nate, 2007). Our results show that CYFIP2 correlates with three domains related to negative symptoms, blunted affect, passive/apathetic social withdrawal, and lack of spontaneity and flow of conversation, suggesting that altered CYFIP2 expression in DLPFC could participate in the abnormal behavior related to negative symptoms in SZ.

CYFIP2 belongs to the CYFIP family which has been related to autism and intellectual disability (Abekhoukh and Bardoni, 2014). However, the function of CYFIP2 in psychiatric disorders is not clear. CYFIP2 is highly expressed in the cortex brain (Zhang et al., 2019) CYFIP2 participates in the regulation of actin dynamic which is essential for the biological processes related to neurodevelopment such as axonal migration, branching and synapse morphology (Spence and Soderling, 2015) Here we found that CYFIP2 was known to be highly expressed in both excitatory and inhibitory neurons based on the information available for single cell analysis in the Human Protein Brain Atlas (Uhlén et al., 1979). The expression of the CYFIP2 is detected in layers two and three of the prefrontal cortex (Zhang et al., 2020). In the context of SZ, decreased number of neurons has been identified in the layer three of DLPFC (van Berlekom et al., 2020). Besides, a study in the BA46 in DLPFC found morphological alterations in the pyramidal cells, however, the authors did not find an alteration in the spatial organization of pyramidal cells, suggesting a normal neuronal migration in SZ subjects (Larsen et al., 2022)In agreement with our study, CYFIP2 has also been found downregulated in a proteomic study in the cingulate anterior (Föcking et al., 2015). However, the relation between CYFIP2 negative symptoms has not been studied in SZ. Thus, our study provides evidence that CYFIP2 is altered in prefrontal cortex with a possible impact on morphological alterations of pyramidal neurons in layers two and three in schizophrenia and the presence of negative symptoms. Further studies are needed to explore CYFIP2 as a potential target for the development of therapeutic interventions to compensate negative symptoms in schizophrenia related to morphological changes on pyramidal neurons.

### 4.3 Proteomic signature in the dorsolateral prefrontal cortex

The unsupervised hierarchical clustering analysis revealed that the proteomic profile detected for each subject did not allow the segregation between controls and SZ samples. Similar segregation pattern between SZ subjects and controls has been observed in other “omics” gene expression studies of postmortem prefrontal cortex (Santarelli et al., 2020) suggesting that changes in different neuron subpopulations in prefrontal cortex could be responsible of this mixed pattern. In this context, a recent study suggests that SZ could be categorized into two sub-biotypes at a molecular level depending on the DLPFC transcriptome, subset 1: similar DLPFC transcriptome to the unaffected subjects and subset 2: different DLPFC transcriptome to the unaffected subjects (Bowen et al., 2019). In our study, although no segregation between SZ and unaffected subject groups was found, a subgroup of SZ samples classified within the control group while another subgroup of 11 samples segregated from hierarchical clusters of unaffected subjects with the exception of two controls (#C4 and C#10) (Figure 2B) suggesting that maybe the proteomic signature could also allow to discriminate between two subtypes of SZ subjects. In this line, a second meta-analysis study from postmortem DLPFC found that a subset of SZ subjects showed differential expression of inflammatory molecules in comparison to another subset of SZ subjects and to unaffected subjects (Childers et al., 2022). Our results showed a limited set of proteins involved in the immune response that could be related to a sub-set of SZ subjects that are susceptible to the proinflammatory cytokines due to the aberrant molecular mechanism of the immune response. Alternately, the mixture presence of two SZ subtypes could explain why there were a reduced number of altered proteins detected.

### 4.4 Limitations

The use of human postmortem brain constitutes a useful tool to dissect the molecular pathways disrupted in psychiatric disorders. However, it has limitations. First, confounding factors including age, postmortem delay, and pH must be carefully explored. In our study, we did not find any association between these variables and the significantly altered proteins in the DLPFC. Second, the possible effect of laterality in our samples cannot be ruled out since all of SZ samples were from left hemisphere. However, we did not find any difference in the proteomic profile when comparing 10 right and 10 left hemisphere samples of control healthy individuals. Third, patients with chronic schizophrenia had been treated with long-term, heterogeneous antipsychotics medications. However, we did not find any significant association with the chlorpromazine equivalent doses. Fourth, our study only included men, who do not represent a real population of this disorder. There was no brain sample availability of females for SZ group in this biobank. Future studies including both genders are needed to elucidate the proteomic changes in females compared to males. Fifth, our study includes samples from different brain biobanks, however, no substantial differences in clustering analysis were observed between centers. Sixth, our study includes institutionalized SZ subjects and non-institutionalized unaffected subjects. The lowest social contact of SZ subjects institutionalized could contribute to the cognitive alteration (Maia, 2010). A study has shown that differences in subcortical alterations between institutionalized and non-institutionalized (Zhao et al., 2022) However, the differences in the cortical neuronal circuits and behavior between SZ subjects institutionalized or not remain poorly understood. Therefore, further studies with both groups institutionalized would be of interest. Finally, our study included elderly individuals due to the type of sample available.

## 5 Conclusion

Our results in dorsolateral prefrontal cortex showed enriched pathways that involved proteins related to MHC class II antigen presentation, immune system and transport. Furthermore, the enriched pathways were integrated into a unique immune response module in the network analyses. Also, our results suggest that RAB7A could have a central role in the immune response due to its interaction with proteins of all the enriched pathways. Thus, our proteomic study provides a possible dysfunction in the immune response in dorsolateral prefrontal cortex in schizophrenia, suggesting that this region could be an area susceptible to damage in schizophrenia subjects due to a failure in immune protection in this area. These results could provide a set of proteins related to dysfunction in the immune response or involved in negative symptoms in schizophrenia.

## Data Availability

The datasets presented in this study can be found in online repositories. The names of the repository/repositories and accession number(s) can be found in the article/Supplementary Material.

## Abbreviations

ACTR1A, Alpha-centractin; AP2M, AP-2 complex subunit mu; CYFIP2, Cytoplasmic FMR1-interacting protein 2; DLPFC, Dorsolateral prefrontal cortex; FDR, False Discovery Rate; FAB, Frontal Assessment battery; LC/MS-MS, Liquid chromatography-tandem mass spectrometry; LFQ, Level free quantification; MHC II, Major histocompatibility complex class II; SZ, Schizophrenia; RAB7A, Ras-related protein Rab-7a.
