# Supplementary material for: Analysis of networks in the dorsolateral prefrontal cortex in chronic schizophrenia: Relevance of altered immune response
Source: Front Pharmacol. 2023 Mar 23;14:1003557. doi: 10.3389/fphar.2023.1003557 (PMC10076656; doi:10.3389/fphar.2023.1003557)
Supplement: Supplementary file 3 [file Table1.docx]

Supplementary Material


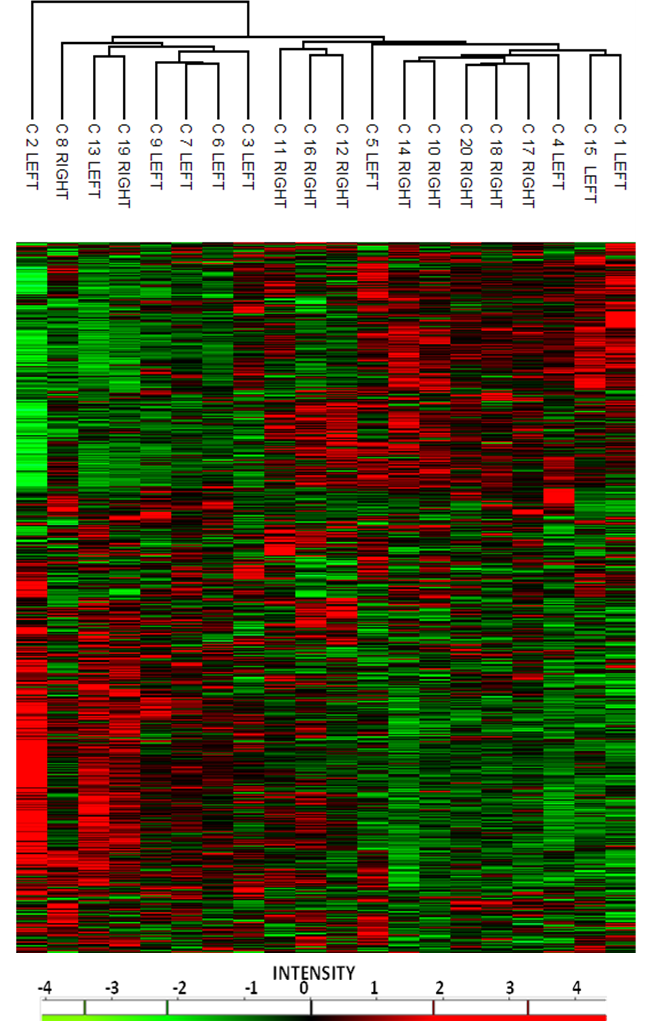


***1 3 1 3 2 2 1 1 3 3 3 1 3 3 3 3 3 1 2 1***

**Figure S1.** **Laterality analysis between the right and left hemispheres from healthy controls**. Unsupervised hierarchical clustering shows laterality analysis between the right and left hemispheres from healthy controls for quantified proteins of 10 right hemispheres and 10 left hemispheres from postmortem prefrontal cortex of healthy control samples. Green color clusters represent downregulated proteins. Red color clusters represent upregulated proteins. The italic numbers indicate the biobank origin of the brain sample: *1* for Navarra Biomed Biobank, *2* for Hospital Clinic IDIBAPS Biobank and *3* for the Institute of Neuropathology of Hospital *Universitari de Bellvitge*.
